# MetNC: Predicting Metabolites *in vivo* for Natural Compounds

**DOI:** 10.3389/fchem.2022.881975

**Published:** 2022-05-12

**Authors:** Zikun Chen, Deyu Yan, Mou Zhang, Wenhao Han, Yuan Wang, Shudi Xu, Kailin Tang, Jian Gao, Zhiwei Cao

**Affiliations:** ^1^ Dept. of Gastroenterology, Shanghai Tenth People’s Hospital, School of Life Sciences and Technology, Tongji University, Shanghai, China; ^2^ International Human Phenome Institutes, Shanghai, China; ^3^ Department of Thoracic Surgery, Fudan University Shanghai Cancer Center, Shanghai, China; ^4^ School of Life Sciences, Fudan University, Shanghai, China

**Keywords:** natural compounds, *in vivo* biotransformation, metabolites, prediction, reaction rules

## Abstract

Natural compounds (NCs) undergo complicated biotransformation *in vivo* to produce diverse forms of metabolites dynamically, many of which are of high medicinal value. Predicting the profiles of chemical products may help to narrow down possible candidates, yet current computational methods for predicting biotransformation largely focus on synthetic compounds. Here, we proposed a method of MetNC, a tailor-made method for NC biotransformation prediction, after exploring the overall patterns of NC *in vivo* metabolism. Based on 850 pairs of the biotransformation dataset validated by comprehensive *in vivo* experiments with sourcing compounds from medicinal plants, MetNC was designed to produce a list of potential metabolites through simulating *in vivo* biotransformation and then prioritize true metabolites into the top list according to the functional groups in compound structures and steric hindrance around the reaction sites. Among the well-known peers of GLORYx and BioTransformer, MetNC gave the highest performance in both the metabolite coverage and the ability to short-list true products. More importantly, MetNC seemed to display an extra advantage in recommending the microbiota-transformed metabolites, suggesting its potential usefulness in the overall metabolism estimation. In summary, complemented to those techniques focusing on synthetic compounds, MetNC may help to fill the gap of natural compound metabolism and narrow down those products likely to be identified *in vivo*.

## Introduction

Modern drug discovery has benefited from nature (Zhu et al., 2011), with more than 30% of approved drugs being provided by or derived from natural compounds (NCs) (Newman and Cragg, 2007). NCs undergo complex and dynamic biotransformation processes to produce a series of metabolites (Beniddir et al., 2021), part of which may relate to efficacy (Segala et al., 2017), safety (Kloprogge et al., 2018), and adverse reactions (Hughes et al., 2016). Understanding the *in vivo* transformation of NCs may help in new drug research and development (Brunmair et al., 2021). Typically, the biotransformation of NCs is carried out in multiple organs, with the liver as the major one through the enzyme family of cytochrome P450 (CYP450) (Rudolf et al., 2017). Yet, with continuous investigation, this process was found to be highly complex involving digestion (Chen et al., 2009), microbial metabolism (Fan and Pedersen, 2021), and other unknown reactions, in addition to CYP450 metabolism. For instance, partial NCs could be degraded into hydrolysate in the acid gastric environment (Van Den Abeele et al., 2017). Also, diet-derived glycans can be metabolized by intestinal bacteria, with one frequently reported as *Bifidobacterium* that belongs to *Actinobacteria*, *Bifidobacteriales* (Milani et al., 2017).

In recent years, extensive technologies have been set up to identify metabolites *in vivo* for NCs, of which those commonly used include liquid chromatography (LC) (Reed and Forgash, 1968), mass spectrometry (MS) (Lisboa and Gustafsson, 1969), or LC-MS (Rodgers et al., 2009). In order to characterize those metabolites, additional chemical reference standards need to be constructed prior to the metabolite’s identification (Witting and Bocker, 2020), covering potential intermediate metabolites of interest as many as possible. Partially due to the aforementioned challenge, the identified *in vivo* metabolites remain deficient in the area of NCs. Meanwhile, computational techniques have been highly desired to generate comprehensive profiling for those likely metabolites *in vivo*. Though no tailor-made algorithm for metabolism prediction has been designed for NC, several articles have tried in this direction to estimate the bio-transformed profiling for chemical compounds. For instance, based on the CYP450 enzyme family, a notable method of GLORY (de Bruyn Kops et al., 2019)/GLORYx (de Bruyn Kops et al., 2021) was developed to predict the oxidation, reduction, and hydrolysis as well as conjugation reactions in the liver for synthetic compounds. Furthermore, handy software, BioTransformer, was set up to predict small molecule metabolism in human tissue, the human gut, as well as experimental environment (Djoumbou-Feunang et al., 2019). From their published training and testing data, it can be seen that they are mainly oriented on synthetic compounds. As NCs contain more polycyclic and endocyclic sub-structures than synthetic compounds (Yang, 2005), this unique structural diversity raised the possibility of biotransformation preference to some extent. In other words, metabolic differences may exist between naturally derived and artificially made chemical structures. So, it is necessary to develop an alternative method for NC to complement with the previous ones for synthetic compounds. Meanwhile, the prediction performance of a method was previously evaluated by a single parameter of coverage on a set of testing data, which was defined as the portion of known metabolites that were successfully predicted (de Bruyn Kops et al., 2021). Yet, over-prediction can lead to high coverage and subsequently high false-positives. So, it is also desired to develop additional parameters for an overall evaluation.

Here, we proposed the MetNC method to predict *in vivo* metabolites for NCs. Previously, we collected 850 biotransformation pairs for herbal ingredients (Kang et al., 2013). For each sourcing compound, the metabolizing product was all validated by comprehensive experimental results of mammals. We extensively explored these biotransformation patterns and summarized enzyme-free reaction rules for model construction. Then, the optimal reaction order was derived for different functional groups in NCs. Coupled with further steric hindrance ranking, MetNC can recommend the most likely candidates bio-transformed *in vivo* among those dynamic metabolizing environments. As the structures of NC and their *in vivo* products are often highly diverse, MetNC may help to estimate the potential transformed profile prior to experiments, so as to facilitate the identification of NC metabolism.

## Materials and Methods

### Souring Dataset

The information of natural compound *in vivo* metabolism was collected from the literature (Kang et al., 2013), and there were 850 compound–metabolite pairs of natural product data remained. Both compounds and metabolites comply with the Simplified Molecular Input Line Entry Specification (SMILES) (Daylight Chemical Information Systems, 2022b) format. See Supplementary Table S1 for all records.

### Reaction Rules

RDKit software (Landrum, 2017) was used to create the visualization image that facilitates artificial reading. After multiple identifications, a total of 60 enzyme-free reaction rules were recognized as practical. Subsequently, the curated reaction rules were converted to a programming language according to the SMILES arbitrary target specification (SMARTS) (Daylight Chemical Information Systems, 2022a) format. See Supplementary Table S2 for detailed reaction rules.

### Evaluation Metric

MetNC regarded Coverage and Sorting ability (CS) as an evaluation metric for the metabolism prediction method. The CS consisted of two parameters: coverage (C) and sorting ability (S), and the mathematical expression of CS is expressed as in Eq. 1:

$$CS=100\times \sqrt{C\times S}$$ (1)

Coverage intended the percentage of correctly predicted metabolites in the sourcing dataset, and the mathematical expression of C is expressed as in Eq. 2:

$$C=\frac{Count_{correctly\_known\_metabolites}}{Count_{total\_known\_metabolites}}$$ (2)

Sorting ability intended the rank of the correct metabolite in the list of candidate metabolites, and the mathematical expression of S is expressed as in Eq. 3:

$$S=\frac{1}{n}\sum_{i=1}^{n}\frac{1}{Order_{i}}$$ (3)

### Metabolite Generation

A total of 60 reaction rules were divided into eight groups according to the functional groups, including alkanes, esters, amines, aromatics, alkenes, ethers, alcohols, and the other functional groups. Any natural compound will first identify its main functional group categories for matching reaction in SMARTS. Subsequently, a series of temporary molecules will be generated via the aforementioned chemical equations.

### Ranking Algorithm

Here, the final rank depended on activity sorting and fine-tuning sorting. The eight functional groups produce 40,320 activity patterns via the enumeration method. After high-intensity calculations, the optimal functional group order was considered: esters > ethers > aromatics > others > amines > alkanes > alkenes > alcohols. Fine-tuning sorting mainly involved structural isomerism sorting. A computational method for calculating the steric hindrance of structural isomerism was designed here. Finally, the top 50 candidate molecules will be regarded as the most potential metabolites.

The pseudocode for calculating the steric hindrance is as follows:

Algorithm 1 Pseudocode for calculating the steric hindrance.

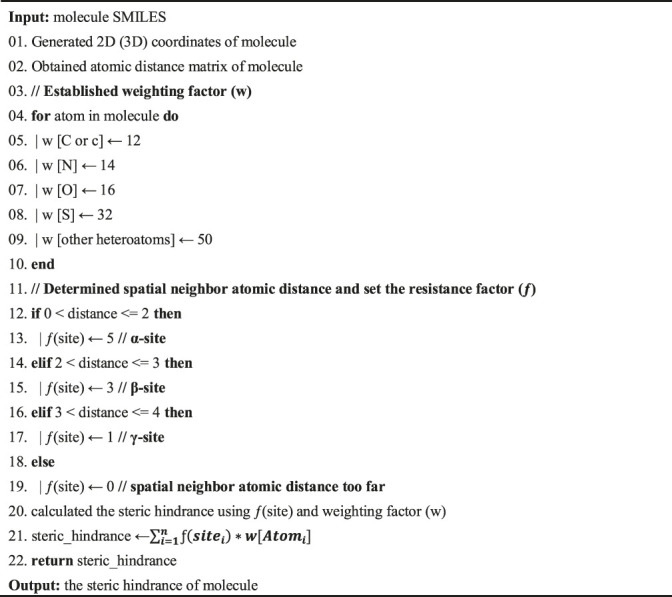

## Results

### Design Principles of MetNC

**FIGURE 1:**
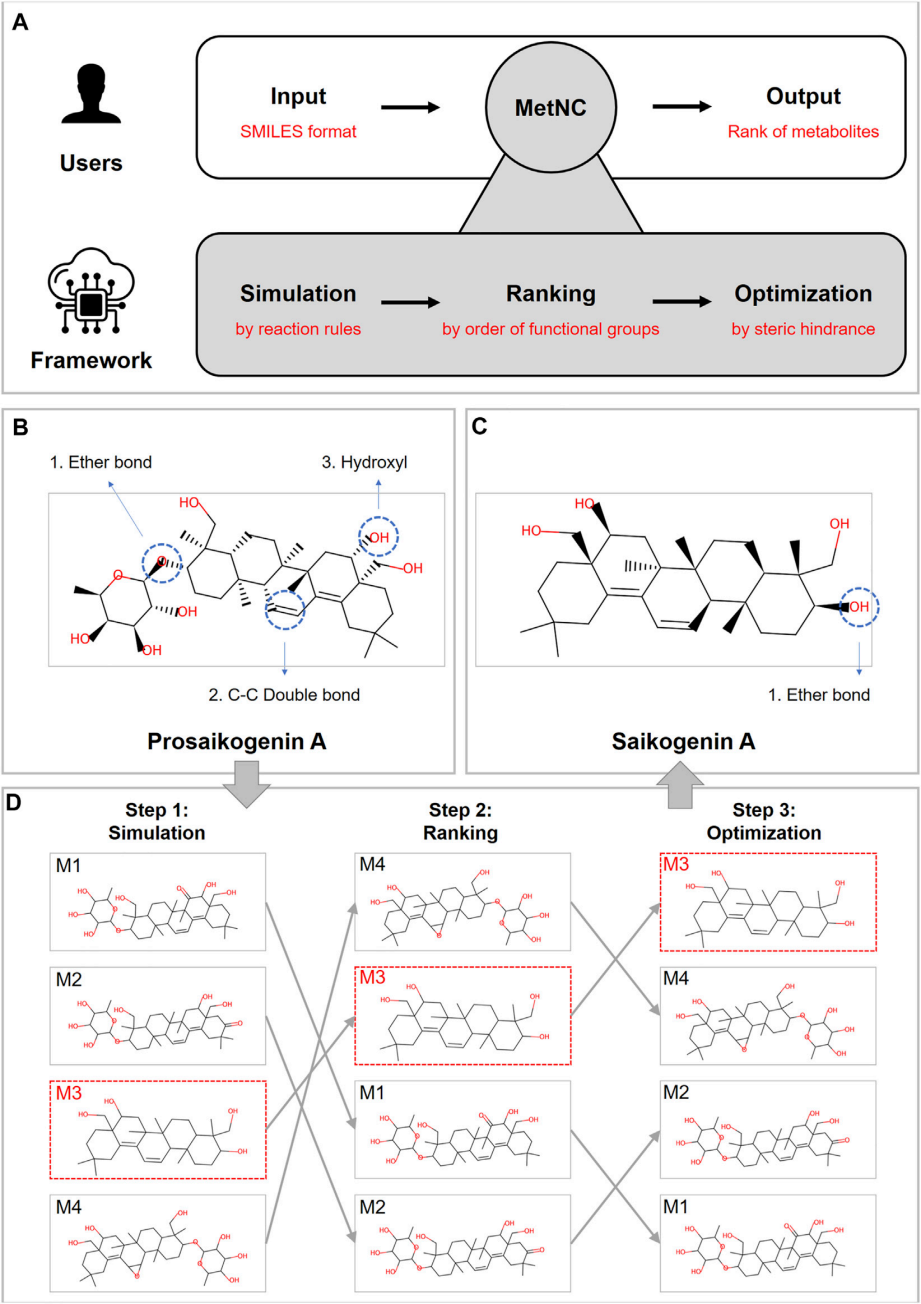
**(A)** Design principles of MetNC. **(B)** Structure information of prosaikogenin A. **(C)** Structure information of saikogenin A. **(D)** MetNC computational flow of prosaikogenin A to predict saikogenin A.

MetNC provides a simple and easy-to-use workflow (Figure 1A): users only need to input the SMILE format of parent NCs, and a ranked list of potential metabolites will be generated. Inside, MetNC was constructed by a three-layer algorithm to gradually derive the resulting list.

First, a collection of potential metabolites will be generated through simulating reactions rules summarized from the sourcing dataset (detailed rules displayed in Supplementary Table S2). So far, this dataset includes 850 substrate–product pairs, representing the largest record of *in vivo* metabolite for NCs. Second, a ranking algorithm was applied to the aforementioned set of metabolites according to the reaction priority of eight functional groups (esters > ethers > aromatics > others > amines > alkanes > alkenes > alcohols). At last, the optimal list was provided based on steric hindrance, and MetNC recommends the top 50 metabolites as a default for each input compound.

Taking prosaikogenin A as an example (Figure 1B), though it was reported with significant effect on anti-platelet aggregation *in vitro* (Zeng et al., 2016), it was found to be transformed into saikogenin A (Figure 1C) before entering blood (Kida et al., 1998). Theoretically, prosaikogenin A may have multiple reaction sites, such as ether bonds, carbon–carbon double bonds, and hydroxyl groups (Figure 1B). So, in step 1, a pool of candidate products was generated after metabolism simulation, by considering all reaction possibilities. Furthermore, in step 2, potential products were ranked according to functional groups, in which those from ether bond breaking were pushed to the top ranking position. In the last step, the ranking list was further sorted via steric hindrance of structural isomerism, so that, the true product in red-dotted frame can be prioritized to the top few (Figure 1D).

### Performance of MetNC on Sourcing Data

In this article, in addition to the parameter of coverage, it is also desirable to evaluate the sorting ability of candidate metabolites for each method, which was simply illustrated by the reciprocal of the ranking position for the known metabolite in the prediction list. Here, we define a new parameter (CS) for overall method assessment on a testing dataset through multiplying the coverage and sorting ability. The range of CS is 0–100 (see Methods for details). The higher the score, the better the performance.

**FIGURE 2:**
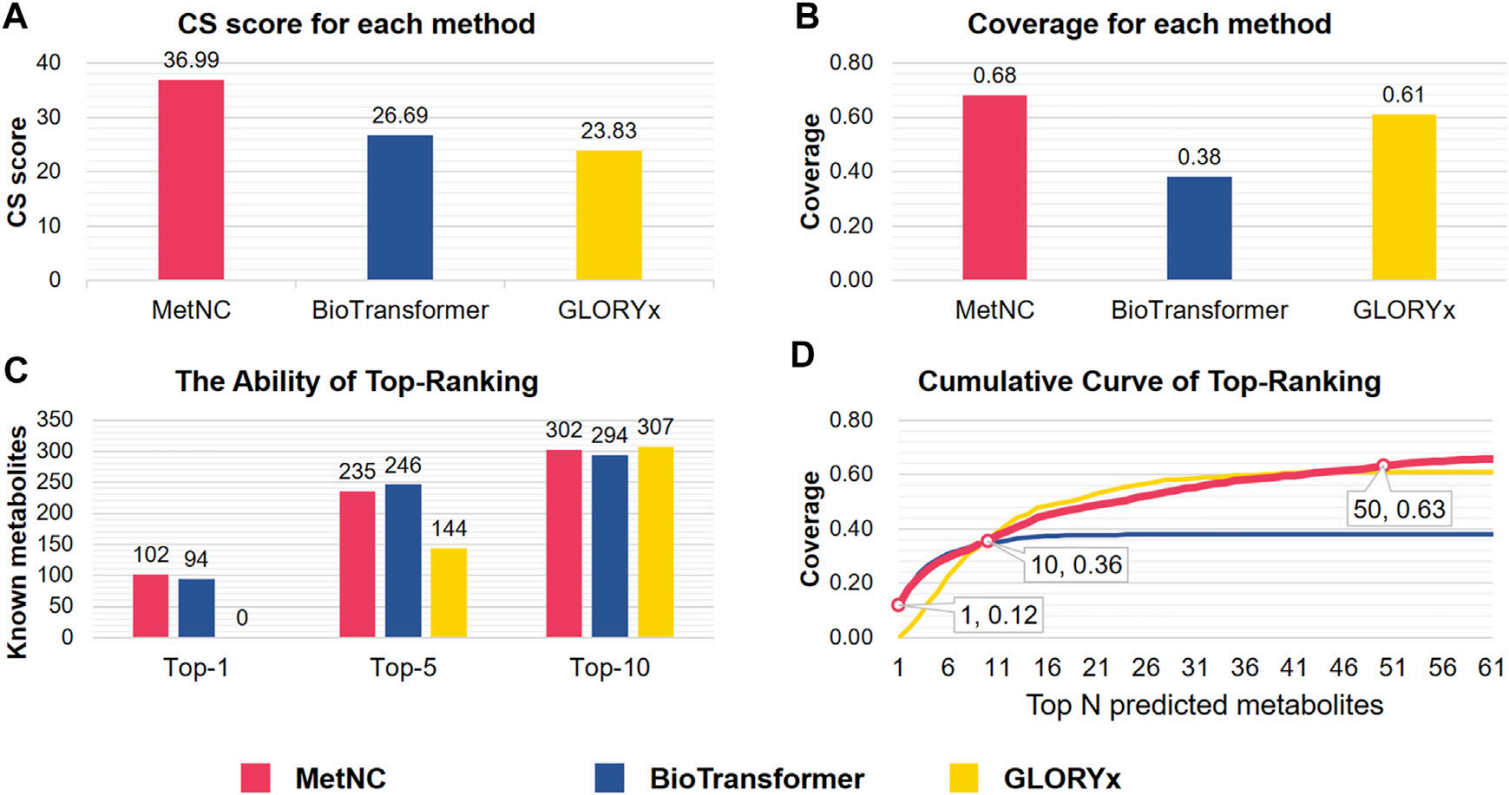
Performance comparison of MetNC, BioTransformer, and GLORYx on the sourcing dataset (*n* = 850). **(A)** CS of each method. **(B)** Prediction coverage of each method. **(C,D)** Ability of ranking known metabolites into the Top-N list. **(D)** Abscissa represents the accumulation from Top-1 to Top-N, while the ordinate indicates accumulative coverage.

Together with peers of BioTransformer and GLORYx, MetNC was tested on 850 substrate–product pairs of the sourcing dataset in terms of CS. As Figure 2 indicated, MetNC gave the highest CS of 36.99, and BioTransformer achieved second with a CS of 26.69 followed by GLORYx with a CS of 23.83 (Figure 2A). Further breakup showed that 578 out of 850 products can be successfully captured by MetNC with the highest coverage of 0.68 (Figure 2B). The top-ranking ability was also illustrated in Figure 2C, indicated by the number of true positives among Top-N predictions for each method. It can be seen that BioTransformer indeed gave nice ranking for the true positives, particularly in the top five list. Yet, overall, only 324 out of 850 (coverage of 0.38) were successfully predicted. Meanwhile, for GLORYx, a higher coverage of 0.61 with 518 successful predictions was achieved, but none of them were ranked into Top-1. In contrast, MetNC ranked 12% of known metabolites in the Top-1 list with the highest-ranking ability and overall coverage. Figure 2D displays the comprehensive ability to push known metabolites into the top-ranking list by the cumulative curve in different ranges of Top-N predictions. In conclusion, MetNC showed the best performance among peering methods on the sourcing dataset.

### MetNC on the Independent Dataset

In total, 14 additional cases were curated from literatures as an independent dataset. Also, the biotransformation of these natural compounds is shown in Supplementary Table S3. Table 1 summarizes the predicted ranking among different methods. It can be seen that MetNC recommended 6 of the 14 known metabolites to the Top-1 list, while BioTransformer only recommended 5, and GLORYx recommended none (Table 1). Of the 14 cases, BioTransformer failed to give prediction on 7 NCs, while both GLORYx and MetNC failed to give on 5. The overall CS reached 56.60 for MetNC, much higher than the second BioTransformer of 46.29.

**TABLE 1:** Ranking performance of three methods on the independent dataset

No.	Prototype compound	Ranking position of the true metabolite			Bio-microbes mediated	Ref.
		MetNC	BioTransformer	GLORYx		
1	Saikosaponin A	1	1	2	Unclear	Liu et al (2013)
2	Saikosaponin B1	1	1	2	Unclear	Yang (2005)
3	Glycyrrhizin	1	1	8	Unclear	Yang (2005)
4	Glycyrrhetic acid 3-O-glucuronide	1	1	25	Yes	Yang (2005)
5	Prosaikogenin F	1	2	10	Unclear	Liu et al (2013)
6	Prosaikogenin A	1	2	18	Yes	Yang (2005)
7	Neoandrographolide 1	2	1	23	Unclear	Yang (2005)
8	Ginsenoside 1	3	Null	45	Yes	Bae et al (2002)
9	Baicalin 1	7	Null	Null	Yes	Zuo et al (2002)
10	Baicalin 2	Null	Null	Null	Yes	Zuo et al (2002)
11	Ginsenoside 2	Null	Null	Null	Yes	Bae et al (2002)
12	Andrographolide	Null	Null	Null	Unclear	Yang (2005)
13	Neoandrographolide 2	Null	Null	Null	Unclear	Yang (2005)
14	Glycyrrhetic acid	Null	Null	5	Yes	Yang (2005)
Overall CS		56.60	46.29	26.99	—	—

**FIGURE 3:**
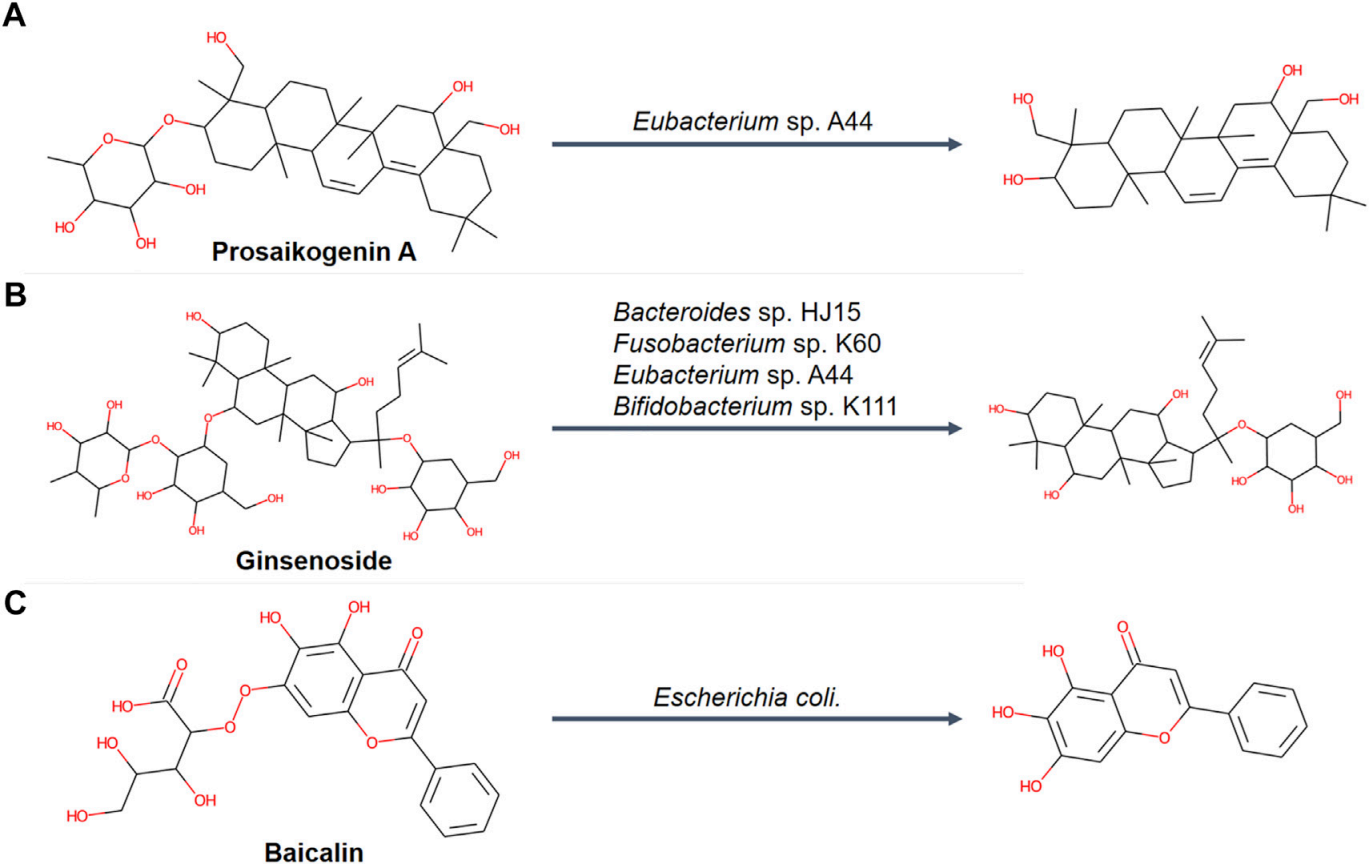
Representative deglycosylation processes mediated by intestinal flora in an independent dataset (*n* = 14). **(A)** Biotransformation of prosaikogenin A that was metabolized by *Eubacterium* sp. A44. **(B)** Biotransformation of ginsenoside that was metabolized by *Bacteroides* sp. HJ15, *Fusobacterium* sp. K60, *Eubacterium* sp. A44, and *Bifidobacterium* sp. K111. **(C)** Biotransformation of baicalin that was metabolized by *Escherichia coli*.

It was noticed that MetNC performed significantly better on prosaikogenin A, ginsenosides, and baicalin than GLORYx and BioTransformer (Table 1, Nos. 6, 8, and 9). Careful investigation found that their metabolism was all commonly reported to be involved by intestinal flora (Figures 3A–C). For instance, prosaikogenin A, an active ingredient from *Bupleurum chinense* DC., was reported to undergo *in vivo* metabolism by an intestine bacterium of *Eubacterium* sp. A44 to the more blood-accessible molecule saikogenin A (Figure 3A) (Kida et al., 1998; Yang, 2005). Also, ginsenoside, a critical component in *Panax ginseng* C.A. Meyer was reported to be metabolized by intestinal flora (Figure 3B), with some known bacteria of *Bacteroides* sp. HJ15, *Fusobacterium* sp. K60, *Eubacterium* sp. A44, and *Bifidobacterium* sp. K111 (Bae et al., 2002). A similar case can be found for the famous compound baicalin, which was converted to baicalein (Figure 3C) via the hydrolysis of *Escherichia coli* before entering the blood circulation (Zuo et al., 2002; Gong et al., 2020). This reminded us to examine all the other compounds with metabolism medicated by bio-microbes. As Table 1 illustrated, seven compounds were found with positive evidence, among which BioTransformer successfully predicted two, while MetNC made four hits. This seems to suggest a unique ability of MetNC to predict metabolites mediated by bio-microbes.

### Optimized Algorithm Improved MetNC Performance

In this article, a three-layer algorithm was constructed for the desired results, including reaction simulation by expert rules, ranking, and further optimization by structural features. Generally, in the area of bio-prediction, machine learning techniques are often applied to large datasets, while on the condition of small datasets, expert rules have shown promising results (Slagle et al., 1984; Tsumoto and Tanaka, 1995). With respect to our relatively small and representative dataset, we summarized 60 general rules regardless of metabolizing organs or environments. Through this, *in vivo* metabolism was initially simulated for each parent compound to produce a candidate list.

When constructing the expert rules, we found that different functional groups attaching to the same structure have different metabolizing preferences. Then, the order of reaction priority was investigated for the eight major functional group categories and incorporated into the algorithm (alcohols, alkanes, alkenes, amines, aromatics, esters, ethers, and others). Meanwhile, for those compounds with the same functional groups, steric hindrance may affect the reaction orders (Jeppsson et al., 1975), which was also taken into consideration in our algorithm.

**FIGURE 4:**
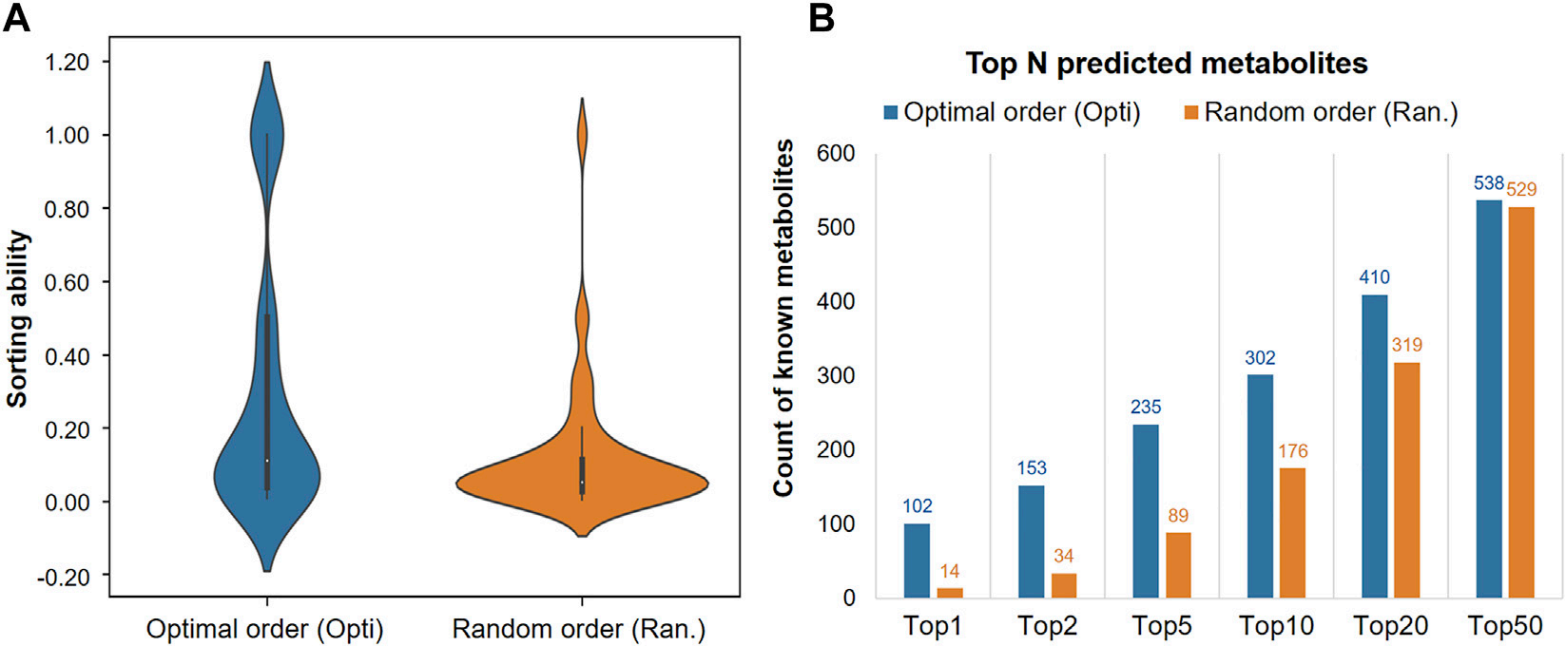
Prioritizing ability of MetNC on different orders of functional groups. **(A)** Ranking position distribution of known metabolites on different orders of functional groups (*n* = 850). **(B)** Number of known metabolites in the Top-N prediction list on different orders of functional groups.

The order of eight functional groups was optimized as: “esters > ethers > aromatics > others > amines > alkanes > alkenes > alcohols”, with Figure 4A showing the significant improvement, compared to a random ordering, such as “alcohols > others > alkanes > esters > aromatics > amines > alkenes > ethers” (student’s *t-test*, *p* = 9.42e-24). Particularly in the top few hits, the optimized order successfully predicted 102 metabolites into Top-1 on the sourcing dataset, while the random order only had 14 successful predictions, as shown in Figure 4B. Various indications show that the MetNC method holds an outstanding ability to predict correct metabolites *in vivo* and also pushes the true metabolite into the top-ranking positions for NCs.

## Discussion

Due to the inherent structural diversity, nature-derived compounds and their metabolites have been re-introduced into therapeutic perspectives in recent years (Thoppil and Bishayee, 2011; Kumar et al., 2013; Zubair et al., 2020). As the structure of one natural compound may contain multiple active sites in different functional sub-groups, it may be metabolized into different products in various *in vivo* microenvironments. In fact, the study of NC biotransformation is just starting, and the identification of likely products remains too challenging and costly. In this article, we constructed a tailor-made method, MetNC, to predict the *in vivo* metabolites based on reaction simulation and candidate sorting, giving the best performance with an extra advantage on the microbiota-mediated metabolism.

On one hand, the high performance of MetNC benefits from the rich and representative dataset of 850 NC metabolism pairs, mainly validated via chromatographic experiments (Kang et al., 2013), from which a set of concise reaction rules can be summarized and applied further to our method. Instead of specific metabolism conditions such as CYP450 or sulfotransferases (SULTs), our reaction rules ignored the detailed organs or enzymes but focused on the overall transformation from substrate to metabolites detected *in vivo*. Another important contribution to performance may lie on the subsequent sorting according to functional sub-groups with their priority ordering. To the end, MetNC significantly improved the ranking of known metabolites by considering not only the site of the metabolizing reaction but also the chemical microenvironment, including chemical activity of functional groups and steric hindrance around the reaction sites.

Among the peers, GLORYx was modeled based on a huge dataset and three sets of more than 200 reaction rules, leading to excellent performance in terms of coverage. Those rules are mainly involved in liver metabolism, covering at least 145 SyGMa’s and 61 GLORY’s rules. On top of that, a new set of GSH conjugation rules augmenting SyGMa’s phase 2 was purposely incorporated to improve the coverage rate but at the cost of precision, as being claimed by their article (de Bruyn Kops et al., 2021). On the other hand, BioTransformer was trained from thousands of data with 237 reaction rules and produced a beautiful ranking for candidate metabolites. Their outstanding raking ability was related to a simple filtering module to eliminate trivial non-candidates at the expense of coverage (Djoumbou-Feunang et al., 2019). While in this article, MetNC summarized a concise set of 60 rules for NC metabolism, successfully combining both their advantages of high coverage and accurate ranking.

Responding to different environmental stimuli, living organisms produce various secondary metabolites with structural diversity and scaffold novelty to defend themselves. As NCs have excellent pharmacological activity and biocompatibility, investigating their biotransformation has become indispensable to seek potent druggable molecules. Yet, the current study was mainly reliant on experiments. Here, MetNC was proposed aiming to estimate the metabolized product profiles for any sourcing natural ingredients, prior to experiments. Using concise reaction rules and rational sorting, it can provide competitive prediction results for NCs. Also, it seems good at not only simulating liver metabolism but also bio-catalyzing via the digestive flora from a holistic perspective. Please be reminded that MetNC was trained by sourcing ingredients from medicinal plants. Compounds from other organisms, particularly with novel scaffolds, may not achieve the best results. In future, MetNC will be improved by the expanded reaction rules and the reaction types not covered in the known dataset.

## Data Availability

The original contributions presented in the study are included in the article/Supplementary Material, further inquiries can be directed to the corresponding authors.

## References

[B1] Bae E.-A. Han M. J. Choo M.-K. Park S.-Y. Kim D.-H. (2002). Metabolism of 20(S)- and 20(R)-ginsenoside Rg3 by Human Intestinal Bacteria and its Relation to *In Vitro* Biological Activities. Biol. Pharm. Bull. 25 (1), 58–63. 10.1248/bpb.25.58 11824558

[B2] Beniddir M. A. Kang K. B. Genta-Jouve G. Huber F. Rogers S. van der Hooft J. J. J. (2021). Advances in Decomposing Complex Metabolite Mixtures Using Substructure- and Network-Based Computational Metabolomics Approaches. Nat. Prod. Rep. 38 (11), 1967–1993. 10.1039/d1np00023c 34821250 PMC8597898

[B3] Brunmair J. Gotsmy M. Niederstaetter L. Neuditschko B. Bileck A. Slany A. (2021). Finger Sweat Analysis Enables Short Interval Metabolic Biomonitoring in Humans. Nat. Commun. 12 (1), 5993. 10.1038/s41467-021-26245-4 34645808 PMC8514494

[B4] Chen C.-Y. Asakawa A. Fujimiya M. Lee S.-D. Inui A. (2009). Ghrelin Gene Products and the Regulation of Food Intake and Gut Motility. Pharmacol. Rev. 61 (4), 430–481. 10.1124/pr.109.001958 20038570

[B5] Daylight Chemical Information Systems (2022a). SMARTS Tutorial. Retrieved from: https://www.daylight.com/dayhtml_tutorials/languages/smarts/index.html .

[B6] Daylight Chemical Information Systems (2022b). SMILES Tutorial. Retrieved from: https://www.daylight.com/dayhtml_tutorials/languages/smiles/index.html .

[B7] de Bruyn Kops C. Šícho M. Mazzolari A. Kirchmair J. (2021). GLORYx: Prediction of the Metabolites Resulting from Phase 1 and Phase 2 Biotransformations of Xenobiotics. Chem. Res. Toxicol. 34 (2), 286–299. 10.1021/acs.chemrestox.0c00224 32786543 PMC7887798

[B8] de Bruyn Kops C. Stork C. Šícho M. Kochev N. Svozil D. Jeliazkova N. (2019). GLORY: Generator of the Structures of Likely Cytochrome P450 Metabolites Based on Predicted Sites of Metabolism. Front. Chem. 7, 402. 10.3389/fchem.2019.00402 31249827 PMC6582643

[B9] Djoumbou-Feunang Y. Fiamoncini J. Gil-de-la-Fuente A. Greiner R. Manach C. Wishart D. S. (2019). BioTransformer: a Comprehensive Computational Tool for Small Molecule Metabolism Prediction and Metabolite Identification. J. Cheminform 11 (1), 2. 10.1186/s13321-018-0324-5 30612223 PMC6689873

[B10] Fan Y. Pedersen O. (2021). Gut Microbiota in Human Metabolic Health and Disease. Nat. Rev. Microbiol. 19 (1), 55–71. 10.1038/s41579-020-0433-9 32887946

[B11] Gong X. Li X. Bo A. Shi R.-Y. Li Q.-Y. Lei L.-J. (2020). The Interactions between Gut Microbiota and Bioactive Ingredients of Traditional Chinese Medicines: A Review. Pharmacol. Res. 157, 104824. 10.1016/j.phrs.2020.104824 32344049

[B12] Hughes T. B. Dang N. L. Miller G. P. Swamidass S. J. (2016). Modeling Reactivity to Biological Macromolecules with a Deep Multitask Network. ACS Cent. Sci. 2 (8), 529–537. 10.1021/acscentsci.6b00162 27610414 PMC4999971

[B13] Jeppsson J.-O. Larsson C. Eriksson S. (1975). Characterization of α1-Antitrypsin in the Inclusion Bodies from the Liver in α1-Antitrypsin Deficiency. N. Engl. J. Med. 293 (12), 576–579. 10.1056/NEJM197509182931203 168490

[B14] Kang H. Tang K. Liu Q. Sun Y. Huang Q. Zhu R. (2013). HIM-herbal Ingredients *In-Vivo* Metabolism Database. J. Cheminform 5 (1), 28. 10.1186/1758-2946-5-28 23721660 PMC3679852

[B15] Kida H. Akao T. Meselhy M. R. Hattori M. (1998). Metabolism and Pharmacokinetics of Orally Administered Saikosaponin B1 in Conventional, Germ-free and Eubacterium Sp. A-44-Infected Gnotobiote Rats. Biol. Pharm. Bull. 21 (6), 588–593. 10.1248/bpb.21.588 9657043

[B16] Kloprogge F. Workman L. Borrmann S. Tékété M. Lefèvre G. Hamed K. (2018). Artemether-lumefantrine Dosing for Malaria Treatment in Young Children and Pregnant Women: A Pharmacokinetic-Pharmacodynamic Meta-Analysis. Plos Med. 15 (6), e1002579. 10.1371/journal.pmed.1002579 29894518 PMC5997317

[B17] Kumar S. Sharma S. Chattopadhyay S. K. (2013). The Potential Health Benefit of Polyisoprenylated Benzophenones from Garcinia and Related Genera: Ethnobotanical and Therapeutic Importance. Fitoterapia 89, 86–125. 10.1016/j.fitote.2013.05.010 23685044

[B18] Landrum G. (2017). RDKit: Open-Source Cheminformatics Software. Retrieved from: http://www.rdkit.org/ .

[B19] Lisboa B. P. Gustafsson J.-Å. (1969). Studies on the Metabolism of Steroids in the Foetus. Biosynthesis of 6α-Hydroxytestosterone in the Human Foetal Liver. Biochem. J. 115 (3), 583–586. 10.1042/bj1150583 5353530 PMC1185141

[B20] Liu G. Tian Y. Li G. Xu L. Song R. Zhang Z. (2013). Metabolism of Saikosaponin a in Rats: Diverse Oxidations on the Aglycone Moiety in Liver and Intestine in Addition to Hydrolysis of Glycosidic Bonds. Drug Metab. Dispos 41 (3), 622–633. 10.1124/dmd.112.048975 23277344

[B21] Milani C. Duranti S. Bottacini F. Casey E. Turroni F. Mahony J. (2017). The First Microbial Colonizers of the Human Gut: Composition, Activities, and Health Implications of the Infant Gut Microbiota. Microbiol. Mol. Biol. Rev. 81 (4), e00036–17. 10.1128/MMBR.00036-17 PMC5706746 29118049

[B22] Newman D. J. Cragg G. M. (2007). Natural Products as Sources of New Drugs over the Last 25 Years. J. Nat. Prod. 70 (3), 461–477. 10.1021/np068054v 17309302

[B23] Reed W. T. Forgash A. J. (1968). Lindane: Metabolism to a New Isomer of Pentachlorocyclohexene. Science 160 (3833), 1232. 10.1126/science.160.3833.1232 4171889

[B24] Rodgers M. A. Saghatelian A. Yang P. L. (2009). Identification of an Overabundant Cholesterol Precursor in Hepatitis B Virus Replicating Cells by Untargeted Lipid Metabolite Profiling. J. Am. Chem. Soc. 131 (14), 5030–5031. 10.1021/ja809949r 19301856 PMC4166558

[B25] Rudolf J. D. Chang C.-Y. Ma M. Shen B. (2017). Cytochromes P450 for Natural Product Biosynthesis in Streptomyces: Sequence, Structure, and Function. Nat. Prod. Rep. 34 (9), 1141–1172. 10.1039/c7np00034k 28758170 PMC5585785

[B26] Segala G. David M. de Medina P. Poirot M. C. Serhan N. Vergez F. (2017). Dendrogenin A Drives LXR to Trigger Lethal Autophagy in Cancers. Nat. Commun. 8 (1), 1903. 10.1038/s41467-017-01948-9 29199269 PMC5712521

[B27] Slagle J. R. Gaynor M. W. Halpern E. J. (1984). An Intelligent Control Strategy for Computer Consultation. IEEE Trans. Pattern Anal. Mach. Intell. PAMI-6 (2), 129–136. 10.1109/tpami.1984.4767498 21869178

[B28] Thoppil R. J. Bishayee A. (2011). Terpenoids as Potential Chemopreventive and Therapeutic Agents in Liver Cancer. Wjh 3 (9), 228–249. 10.4254/wjh.v3.i9.228 21969877 PMC3182282

[B29] Tsumoto S. Tanaka H. (1995). Induction of Expert System Rules Based on Rough Sets and Resampling Methods. Medinfo 8 Pt 1 (Pt 1), 861–865. Retrieved from: https://www.ncbi.nlm.nih.gov/pubmed/8591347 . 8591347

[B30] Van Den Abeele J. Rubbens J. Brouwers J. Augustijns P. (2017). The Dynamic Gastric Environment and its Impact on Drug and Formulation Behaviour. Eur. J. Pharm. Sci. 96, 207–231. 10.1016/j.ejps.2016.08.060 27597144

[B31] Witting M. Böcker S. (2020). Current Status of Retention Time Prediction in Metabolite Identification. J. Sep. Sci. 43 (9-10), 1746–1754. 10.1002/jssc.202000060 32144942

[B32] Yang X.-W. (2005). Key Foundational Science Problem in Experimental Medicine Study of Chinese Materia Medica: Ascertainment of Active and Toxic Constituents from Chinese Materia Medica. J. Chin. Integr. Med. 3 (2), 154–159. 10.3736/jcim20050220 15763068

[B33] Zeng B. Liu G.-D. Zhang B.-B. Wang S.-s. Ma R. Zhong B.-S. (2016). A New Triterpenoid Saponin from Clinopodium Chinense (Benth.) O. Kuntze. Nat. Product. Res. 30 (9), 1001–1008. 10.1080/14786419.2015.1095745 26511166

[B34] Zhu F. Qin C. Tao L. Liu X. Shi Z. Ma X. (2011). Clustered Patterns of Species Origins of Nature-Derived Drugs and Clues for Future Bioprospecting. Proc. Natl. Acad. Sci. U.S.A. 108 (31), 12943–12948. 10.1073/pnas.1107336108 21768386 PMC3150889

[B35] Zubair H. Khan M. A. Anand S. Srivastava S. K. Singh S. Singh A. P. (2022). Modulation of the Tumor Microenvironment by Natural Agents: Implications for Cancer Prevention and Therapy. Semin. Cancer Biol. 80, 237–255. 10.1016/j.semcancer.2020.05.009 32470379 PMC7688484

[B36] Zuo F. Yan M. Z. Zhou Z. M. (2002). Advances in the Study on Metabolism of Effective Constituents of Traditional Chinese Herbal Drugs by Intestinal flora. Zhongguo Zhong Yao Za Zhi 27 (8), 568616–616. 10.3321/j.issn:1001-5302.2002.08.003 Retrieved from: https://www.ncbi.nlm.nih.gov/pubmed/12866503 . 12866503

